# A Rare Tumor in a Patient with Hepatic Hydatic Cyst: Adrenal Hepatoid Adenocarcinoma

**DOI:** 10.1155/2014/824574

**Published:** 2014-02-12

**Authors:** Fatma Umit Malya, Suleyman Bozkurt, Mustafa Hasbahceci, Gokhan Cipe, Issam Cheikh Ahmad, Zuhal Gucin, Oguzhan Karatepe, Mahmut Muslumanoglu

**Affiliations:** ^1^Department of General Surgery, Faculty of Medicine, Bezmialem Vakif University, Vatan Street, Fatih, 34093 Istanbul, Turkey; ^2^Department of Radiology, Faculty of Medicine, Bezmialem Vakif University, Vatan Street, Fatih, 34093 Istanbul, Turkey; ^3^Department of Pathology, Faculty of Medicine, Bezmialem Vakif University, Vatan Street, Fatih, 34093 Istanbul, Turkey

## Abstract

Hepatoid adenocarcinoma (HAC) is a very rare type of extrahepatic adenocarcinoma which has a clinicopathologic and morphologic similarity to hepatocellular carcinoma (HCC). Although it is not common, it can be seen in organs other than the liver. The correct diagnosis can be a challenge because of its clinically similarity to HCC and the diagnosis is usually achieved by pathological examination following the surgery. We present a 48-year-old woman who was following with the diagnosis of stage 5 hepatic hydatic cyst. In her routine blood examinations, her alpha feta protein level was found higher than normal and her abdominal computed tomography and magnetic resonance findings did not reveal any pathological findings rather than hepatic hydatic cysts. There was a high activity of FDG on PET CT in the hepatic region so we performed a right lateral hepatectomy to the patient and final pathology was adrenal hepatoid adenocarcinoma. In this paper we aimed to present a rare case of hepatoid carcinoma of the adrenal gland.

## 1. Introduction

Hepatoid adenocarcinoma (HAC) is a very rare variant of extrahepatic adenocarcinoma [1]. HAC can originate from different organs [2–10]. The stomach is most common site of this tumor. HAC is usually found incidentally in the routine examinations while the patient is followed up for another disease [1]. We aimed to present an asymptomatic case that was found to have a hepatic tumour which had a final pathology as extrahepatic hepatoid adenocarcinoma, while she was following up with stage 5 hepatic hydatic cyst.

## 2. Case Report 

**Figure 1:**
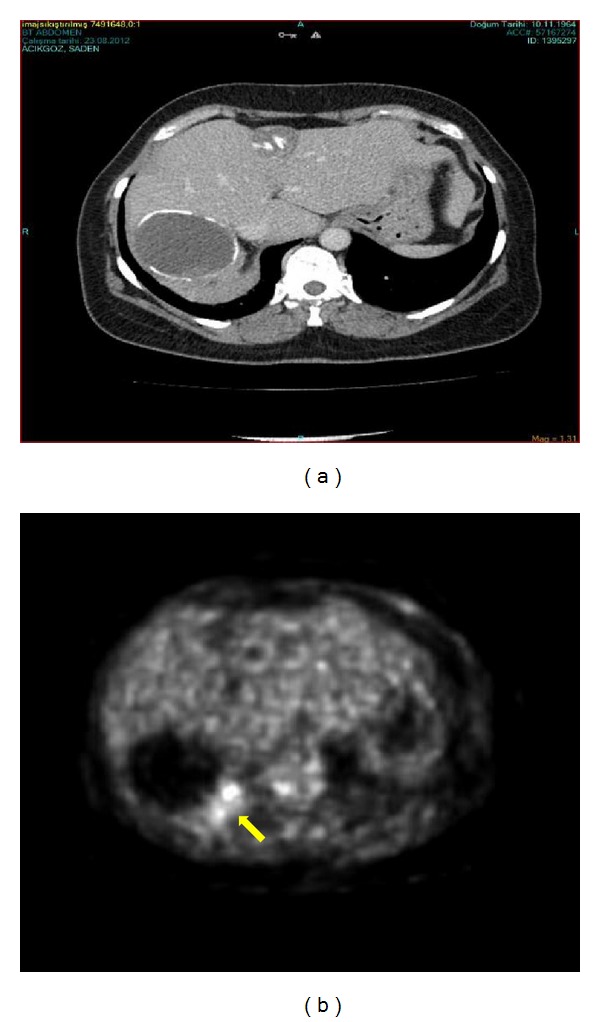
The radiological findings of the patient. (a) Abdominal computed tomography showing a 65 × 55 mm stage 5 hydatic cyst in the right hepatic lobe and a 30 × 28 mm in the left hepatic lobe, (b) FDG18 positron emission tomography of the patient on which the mass revealed a high activity of FDG. Yellow arrow shows the high suvmax activity area (suvmax 80).

**Figure 2:**
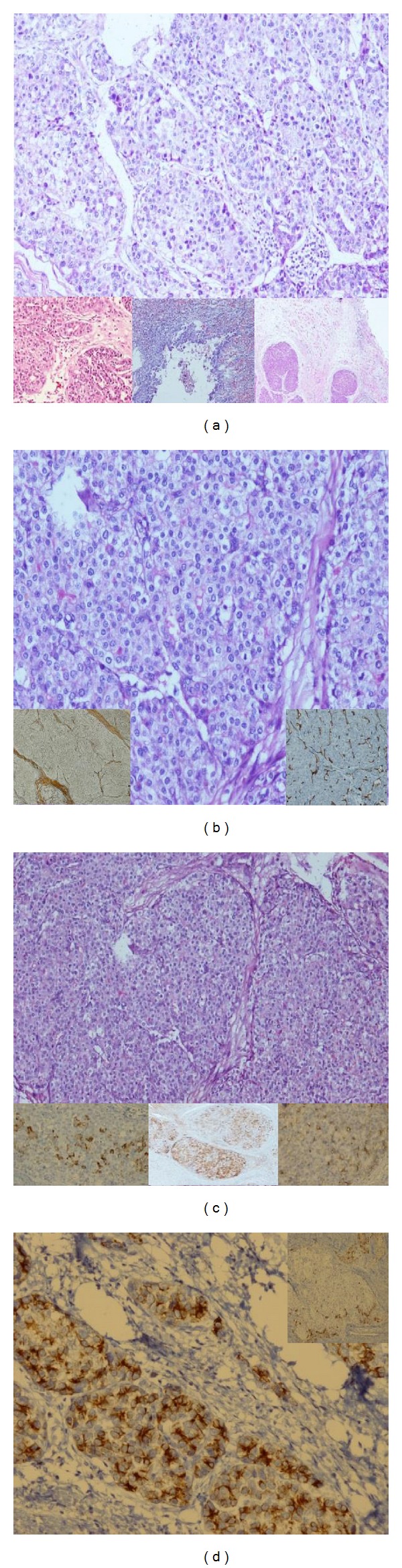
The hystopatological findings of the patient's tumor. (a) The overall view suggested hepatocellular carcinoma (HE ×200) (the big figure). On the below, from left to right we can see the adenoid areas, adrenal invasion, and diaphragmatic invasion (HE ×100, HE ×100, and HE ×40). (b) Thick trabecular structures areas suggesting hepatocellular carcinoma (HE ×200). Reticulin texture, trabecular structures on the left below, and sinusoidal structures with CD34 on the right below (small figures). (c) Her Par1 positivity of tumor cells on the left below (her Par1 ×200); generalized AFP expression of the tumor cells in the middle below (AFP ×100); canalicular type positivity with pCEA on the right below (pCEA ×200). (d) Generalized cytokeratin positivity in the adenoid areas (CK7 ×200) (the big figure); cytokeratin 19 positivity peripherally to the nodule (CK19 ×100) (the small figure on the right above).

We present a 48 years old woman who was following up with the diagnosis of stage 5 hydatic cyst. The serological test is performed for hydatic cyst (indirect hemagglutination test) and the result was 1/640. In her routine examinations alpha feta protein was 3900 n/mL. Abdominal Computed Tomography revealed 65 × 55 mm stage 5 Hydatic cyst in the right hepatic lobe and a 30 × 28 mm in the left hepatic lobe (Figure 1(a)). There was not another pathology detected in abdominal magnetic resonance imaging. In FDG18 Positron Emition Tomography there was a 4 × 5 cm mass in the liver. It was near the right lobe cyst and it was reached to the diaphragmatic crura. This mass revealed a high activity of FDG (suvmax 80) (Figure 1(b)). The patient was operated with the diagnosis of hepatic mass. There was a hydatic cyst in the 6-7th segments of the liver and on the posterior of the cyst there was a 4 × 5 cm mass which was invaded to diaphragmatic crura. Segment 6-7 hepatectomy and diaphragmatic resection were performed. The adrenal glands and paraganglia were normal. The postoperative course was uneventful and the patient was discharged on the fifth day after the surgery. On the hystopatological examination, the tumor was composed of medium-sized polygonal cells with granular eosinophilic cytoplasm resembling to hepatocellular carcinoma. There was not any tumour cells in the liver parenchyma close to the tumour. Therefore immunohistochemical stains were performed to differentiate the origin of the tumor. These tests are used to understand whether the tumor was a real hepatocellular carcinoma or a hepatoid adenocarcinoma ant to differentiate whether the tumor is originated from the liver or from the adrenal gland. The stains showed diffuse +AFP, Glipan, and CK8; +Heppar in the solid areas; +CK17 and 19 in the glandular areas; −chromogranin, CD20, ER, PR, and GCDFP15; +luminal/focal polygonal CEA (Figures 2(a) and 2(b)). It was a trabecular and sinusoidal structure with CD34 and reticulin. Sinaptofisin and inhibin-A were focally positive (Figures 2(c) and 2(d)). Finally the morphologic and immunohistochemical features of the tumour suggested a hepatoid adenocarcinoma originated from adrenal gland. Adjuvant therapy with 5-flourousil and gemcitabine for 6 months and radiotherapy with the dose of 4500 cGy for 5 were decided to be given to the patient.

## 3. Discussion

Hepatoid adenocarcinoma (HAC) is a rare variant of extrahepatic adenocarcinoma which is morphologically similar to hepatocellular carcinoma (HCC) [1]. HAC usually occurs in the sixth or seventh decade of life and is more common in men [1]. In our case the tumor was located on the liver and there was a high serum level of AFP and our initial diagnosis was HCC. HAC can originate from stomach, gallbladder, urinary bladder, colon, ureter, lung, ovary, pancreas, adrenal gland, and peritoneum [2–10]. HAC of urinary tract is very rare. We found two cases of AFP producing tumors originated from adrenal gland. One of them was an adrenocortical adenocarcinoma and the other was an adrenal HAC [1, 6–8, 11]. Following the intensive review of 98 articles from 2001 to 2011, 217 patients of HAC were found and most of these were gastric HAC.

In a review series of 98 articles from 2001 to 2011 there are 217 patients of HAC and most of them were gastric HAC.

Clinical presentation of HAC depends on the anatomic location of the tumor. HAC is usually metastatic in the initial presentation [1]. The most common sites of metastasis were lymph nodes, liver, and lung.

The imaging studies are the basic diagnostic features for HAC. On the computed tomography HAC can be detected as a hypervascular tumor at any of the suggesting organs. HCC up takes contrast in arterial phases and washes out in late series. HAC may show similar pattern on computed tomography or it can be detected on liver as an atypical mass. It is difficult to separate HAC from HCC by this way. Therefore any other atypical mass should be considered in differential diagnosis. Biopsy may be helpful to confirm the diagnosis. In a few cases like in ours, HAC can simultaneously be found with a hemangioma or a hydatic cyst. The HAC of the liver can present with portal vein thrombosis as similar to HCC. On PET CT, elevated suvmax values are related to presence of a malignancy. However HCC and HAC, both, have elevated levels of suvmax values and the differential diagnosis regarding these values is not considered. In our case, the high level of suvmax on PET CT may be due to infection of the cyst, the IHA level, and type of the cyst are not suggesting a severe infection to confuse PET CT findings. Therefore with the light of these findings we can say that if there is an elevated serum level of AFP with a mass detected on an imaging technique, extrahepatic hepatoid adenocarcinoma is a diagnosis which should be considered as a rare cause. Pathological markers and immunohistochemical (IHC) stains provide the definitive diagnosis for HAC. In our case we had a adrenal HAC in the liver. It is suggested that the mass is more likely to be a locally invasive tumor than to be a metastatic lesion.

The only therapeutic approach for HAC is surgery. In general HAC has a poor prognosis. The estimated mean survival is limited to 12 months. There is not a specific adjuvant procedure proven to be effective on survival. The chemotherapeutics which are used for HCC may be helpful.

## 4. Conclusion

Hepatoid adenocarcinoma is a rare type of extrahepatic adenocarcinoma which is clinically and morphologically similar to HCC. When a mass detected on an imaging technique outside the liver with an elevated serum level of AFP, extrahepatic hepatoid adenocarcinoma is a diagnosis which should be considered in such a rare cause. If the routine imaging techniques are not helpful with an elevated serum level of AFP, 18FDG PET scan may be helpful for the correct diagnosis. However, the pathological examination is mandatory for the definitive diagnosis. Finally, during routine followup of patients with a known diagnosis, it has always to be kept in mind that other accompanying pathologies may occur.
